# The usability and feasibility validation of the social robot MINI in people with dementia and mild cognitive impairment; a study protocol

**DOI:** 10.1186/s12888-022-04418-9

**Published:** 2022-12-05

**Authors:** Aysan Mahmoudi Asl, Jose Miguel Toribio-Guzmán, Henriëtte van der Roest, Álvaro Castro-González, María Malfaz, Miguel A. Salichs, Manuel Franco Martin

**Affiliations:** 1 Psycho-Sciences Research Group of IBSAL, Salamanca University, 37007 Salamanca, Spain; 2 Department of Research and Development, Iberian Institute of Research in Psycho-Sciences, INTRAS Foundation, Zamora, Spain; 3 Department on Aging, Netherlands Institute of Mental Health and Addiction (Trimbos Institute), Utrecht, The Netherlands; 4 Robotics Lab, Department of System Engineering and Automation, Universidad Carlos III de Madrid, Av. de la Universidad 30, Leganés, 28911 Madrid, Spain; 5 Psychiatry and Mental Health Service, Assistance Complex of Zamora, Zamora, Spain

**Keywords:** Social robots, Usability, Feasibility, Acceptability, Study design, Dementia

## Abstract

**Background:**

Social robots have demonstrated promising outcomes in terms of increasing the social health and well-being of people with dementia and mild cognitive impairment. According to the World Health Organization’s Monitoring and assessing digital health interventions framework, usability and feasibility studies are crucial before implementing prototype social robots and proving their efficacy and effectiveness. This protocol paper aims to detail the plan for conducting the usability and feasibility study of the MINI robot based on evidence-based recommended methodology.

**Methods:**

In this study, an experimental design and a mixed method of data collection will be applied. Twenty participants aged 65 and over with dementia or mild cognitive impairment will be recruited. Eight sessions of interaction with the robot, as well as qualitative and quantitative assessments, will be accomplished. The research will take place in a laboratory. Ethical approvals have been acquired. This research will be valuable in the development of the MINI robot and its practical deployment in the actual world, as well as the methodological evidence base in the sector of social robots.

**Discussion:**

By the winter of 2022–2023, the findings of this study will be accessible for dissemination. This study will aid to improve the evidence-based methodology used to study the feasibility and usability of social robots in people with dementia and mild cognitive impairment as well as what can be learned to advance such study designs in the future.

**Supplementary Information:**

The online version contains supplementary material available at 10.1186/s12888-022-04418-9.

## Background

According to the Alzheimer Europe Yearbook 2019, about 9.8 million people are living with dementia in Europe, and this number is expected to double by the year 2050, affecting 18.8 million people [1]. According to demographic trends, Spain has one of the highest incidences of dementia, with 6.3% of those over 60 having the disease [2]. Northern European countries such as Norway and Sweden, as well as the United States, Japan, and the United Kingdom, share similar concerns, prompting them to take major steps toward potential solutions. Dementia is one of the most common neurodegenerative diseases causing a deterioration in memory, thinking, behavior, and the ability to perform everyday activities. Limited social interactions and small social networks are associated with the faster progression of dementia mutually and place people with dementia at a higher risk of experiencing adverse events [3]. Dementia causes a significant burden on caregivers and families and affects their health as well [4].

In a search for innovative dementia care strategies, a quite young field of research has been dedicated to social robots for people with dementia (PwD). The social robots are employed to enhance people’s well-being, autonomy, and independence, thus enabling them to live independently for longer [5]. Socially assistive robots (SARs) may be categorized as service robots and companion robots. Companion robots such as Pepper, Nao, Zora, Hobbit, and Aibo have been frequently studied, yielding promising results in psychosocial domains such as improved emotions and mood, engagement, social interactions, participation for elderly adults, and PwD [6]. These interactive robots provide companionship and enhance social interactions, resulting in improved social health. In particular, Paro and Nao excelled in this role in a few studies, reducing feelings of loneliness, and improving mood and social interaction [6–8]. Despite promising results, there is no strong evidence based on the effectiveness of social robots on (neuro) psychosocial outcomes [9]. Thus far, the effectiveness of social robots on cognition and quality of life has not been proven [6]. Also, outcomes such as feelings of loneliness are less investigated compared to other subjective measures [6].

To ensure the efficacy and effectiveness of social robots, we must first determine whether they are perceived as usable and acceptable by potential users. Physical, psychosocial, and behavioral factors of interaction with a social robot have been shown in studies to contribute to the user’s overall acceptance of the robot. The technological features of social robots play an important role in usability measures, and according to Mitzner et al. [10], usability is a perfect predictor of the social robot’s acceptability.

Recently the Robotics Lab from Carlos III University of Madrid developed a plush-like desktop social robot, called ‘MINI ’[11]. MINI aims to support seniors and provide cognitive and social stimulation for older people with neurodegenerative diseases, especially for those with cognitive impairment. The robot can autonomously interact with humans through verbal and nonverbal communication. The MINI robot is a research platform that is not commercialized yet. Previous research on social robots recommended designing and developing social robots and tailoring the user interface and characteristics of the robot, to the needs and preferences of PwD [12, 13]. Furthermore, Bradwell et al. [14] proposed differences in preferences between robot developers and end users and encouraged researchers to involve end users in the design and development of social robots. Hence, we will conduct a trial study on PwD and people with mild cognitive impairment (MCI) to explore the usability and feasibility of MINI, as well as, its effect on the observed emotions during the interaction sessions. In this paper, we describe the protocol that will be followed to conduct the abovementioned trial study.

This testing will help to improve the MINI robots’ technological features and functionalities, software, and user interface. The robot implementation will be feasible for individual implementation in clinics and research settings after the necessary features and functions have been added/ modified, enabling researchers to evaluate its impact and effectiveness in different scenarios and settings. We believe that by triggering positive emotions and reducing negative emotions, MINI will benefit PwD and MCI. The study procedure is based on the Standard Protocol Items: Recommendations for Interventional Trials (Guidelines) [15].

The purpose of this study is to evaluate 1) the usability and feasibility of the MINI robot for PwD and MCI, and 2) the effect of the MINI robot on the observed emotions in a laboratory setting. The current study aims to answer the following questions:

## Methods

### Objectives

The purpose of this study is to evaluate 1) the usability and feasibility of the MINI robot for PwD and MCI, and 2) the effect of the MINI robot on the observed emotions in a laboratory setting. The current study aims to answer the following questions:

1. Is MINI considered feasible and acceptable by PwD and MCI?
2. Is MINI usable for PwD and MCI in a research lab?
3. What are the attitudes of PwD and MCI towards MINI?
4. Does the MINI robot intervention trigger instant positive emotions and reduce negative emotions of PwD and MCI?
5. Are changes or modifications to the MINI robot software and hardware features required to improve its usability and feasibility?

### Design

We will employ an experimental methodology and a mixed-method of data collection in this project, which will last for a month for each participant to accomplish the robot interaction sessions. To address the study’s questions, quantitative and qualitative data will be collected using questionnaires, scales, observations, and interviews. The research team will select potential participants at recruiting sites (*n* = 20) based on exclusion/inclusion criteria. The potential participants and their next of kin will be briefed about the study’s topic and urged to sign a consent form. Primary and secondary outcomes will be evaluated at baseline and immediately following the interaction sessions. The methodology of this experiment is based on a recent scoping review [6] recommended methodology for testing the usability and feasibility of social robots.

### Participants and settings

After the participant’s identification and recruitment, the trial will be carried out by the research group at the research lab of Intras foundation, in Zamora, Spain. We will include people based on inclusion and exclusion criteria.

Inclusion criteria:

- PwD and/or MCI
- Aged 65 and over
- Ability to read and write in Spanish
- Ability to decision making
- MMSE score ≤ 24

Exclusion criteria:

- Unstable medical conditions.

### Theoretical framework

**Fig. 1:**
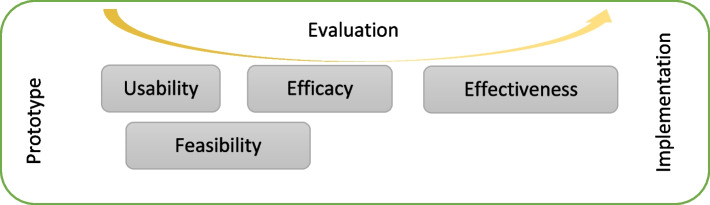
The evaluation and monitoring maturity of the MINI robot over time

The evaluation of the usability and feasibility of MINI will be conducted based on the following models: 1) Monitoring and evaluating digital health interventions framework by the World Health Organization. This framework is intended to guide digital health researchers to understand the different stages of monitoring and implementation of digital health interventions and evaluate them [16]. Based on this framework, the Acceptability study is defined within the feasibility concept. Figure 1 shows the evaluation and monitoring maturity of the MINI robot over time from a prototype to the implementation phase. 2) Almere model; This model is an extension and adaption of the Unified Theory of Acceptance and Use of Technology (UTAUT). In this model, 11 constructs are defined to measure the level of acceptance and usefulness of assistive social agents by elderlies [17]. Besides the functional evaluation of the technology, the Almere model evaluates social interaction-related variables such as perceived sociability and social presence [17].

### Staff training

Before the intervention, all responsible research staff will receive MINI robot master training. In this training, the MINI robot will be presented with its functionalities, software use, put into operation, and switch off maintenance and troubleshooting. They will be responsible for any problem or question regarding the robot’s functionalities. To consistent data collection, the researchers of the Intras foundation (IBIP) will train research staff regarding the method of data collection.

### Pre-experimental phase

This will be carried out in a usability laboratory and users’ interactions with the MINI Robot will be recorded. The users will perform a series of tasks and games in MINI robot for half an hour in one session.

To develop rigorous monitoring of the participants’ interactions and behavior, the interaction sessions will be conducted individually. The sessions will also be audio and video recorded for further review. Each participant may have some prior Information Technology and telecommunications knowledge. Before starting the trial, participants and their next to kin will be asked to sign an informed consent. At the end of the usability session, they will have to complete the acceptability and usability questionnaire which we developed based on the *Almere Model*, an adapted model of the *Unified Theory of Acceptance and Use of Technology*, proposed by Heerink et al. [17] as well as the system usability scale [18].

### Experimental phase

During this phase which will last 1 month, the data will be collected from the participants interacting with the MINI robot. During the sessions, physical observations will be conducted by an independent observer and reported on a record sheet. This data will be analyzed and will serve to assess the problems encountered by users and the difficulties of implementing the robot.

During the interaction sessions, an independent observer will rate the observed affects and emotions using the OERS scale.

### Post-experimental phase

The acceptability and usability questionnaire based on the Almere model will be completed after the trial is over. To gauge the general opinion on the MINI robot interactions, semi-structured interviews will be undertaken. Over the course of a month-long intervention, we will examine the efficacy of the MINI robot in enhancing positive emotions and reducing negative ones. The early, middle and last parts of the intervention period—10 minutes before and 10 minutes during the engagement with the robot—will all involve the observation of emotions.

### Interaction sessions

#### MINI robot

**Fig. 2:**
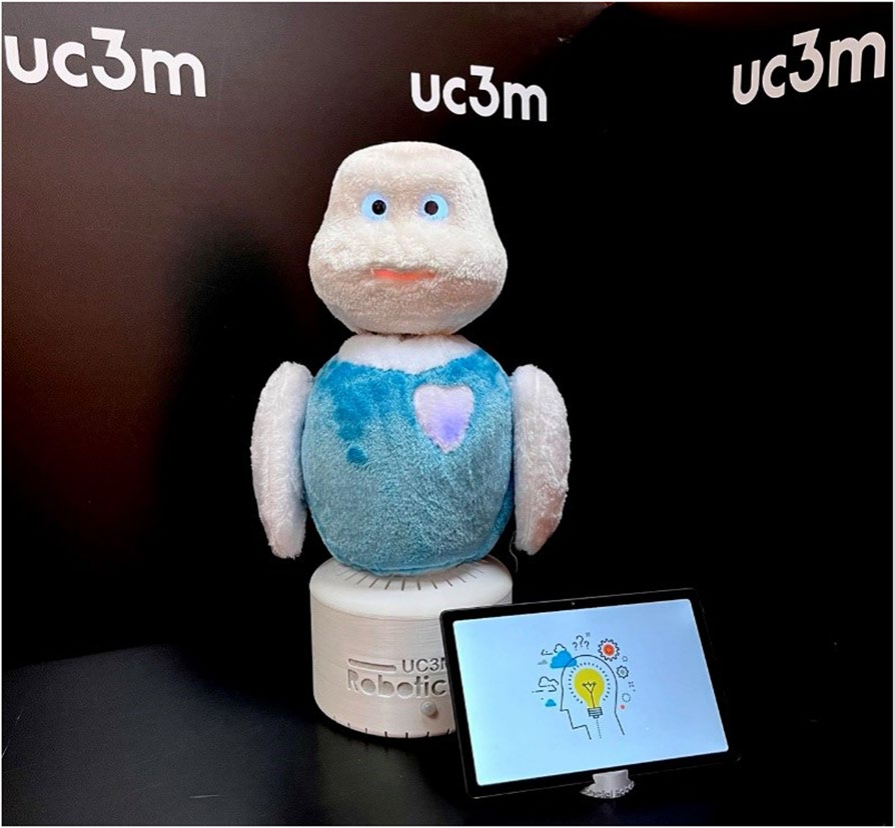
MINI robot

MINI is a social robot specifically designed to assist and accompany elderly people in their daily life either at home or in a nursing facility. The robot can provide psychosocial and cognitive stimulation through mental and cognitive tasks and also provides services in the areas of entertainment and personal assistance. MINI is a lightweight, small-sized, stationary robot with a friendly appearance that is covered in faux fur. It is equipped with a microphone for speech-based communication, touch sensors in the shoulders and belly that allow the user to interact physically with MINI, an RGB-D camera to extract visual and depth information from the environment, uOled eyes, and a tablet that can work both as an input device (through fixed menus) and an output device, displaying photos, videos, music, and other applications. A default series of games and cognitive exercises are installed on the robot that can be extended and modified. MINI is programmed to ask certain questions, process the respond, and provide users with feedback on their performance [19, 20]. Currently MINI is in the prototype phase (See Fig. 2).

### Interaction scenario

For the intervention group, the MINI robot will be applied in the research lab. The intervention duration is a total of 1 month for each participant. The reason to conduct multiple interaction sessions is to eliminate the novelty effect [21] of the MINI robot that might affect the acceptability. The frequency of intervention sessions is two or three times a week based on the participant’s availability, running for half an hour for each participant. An initialization phase before the intervention session will be undertaken, to get the participants familiar with the robot and learn how to interact with it. The user will perform a series of entertainment and memory games while a researcher will be moderating the sessions. MINI will initiate the interaction with the user by touching its shoulder and verbally encouraging them to choose an activity shown on the tablet. The interaction will continue through verbal and non-verbal channels. Six activities will be accomplished in 30 minutes and meanwhile MINI will give feedback as encouragement in case of successful accomplishment of the tasks and guidance in case of repeated failures.

**Fig. 3:**
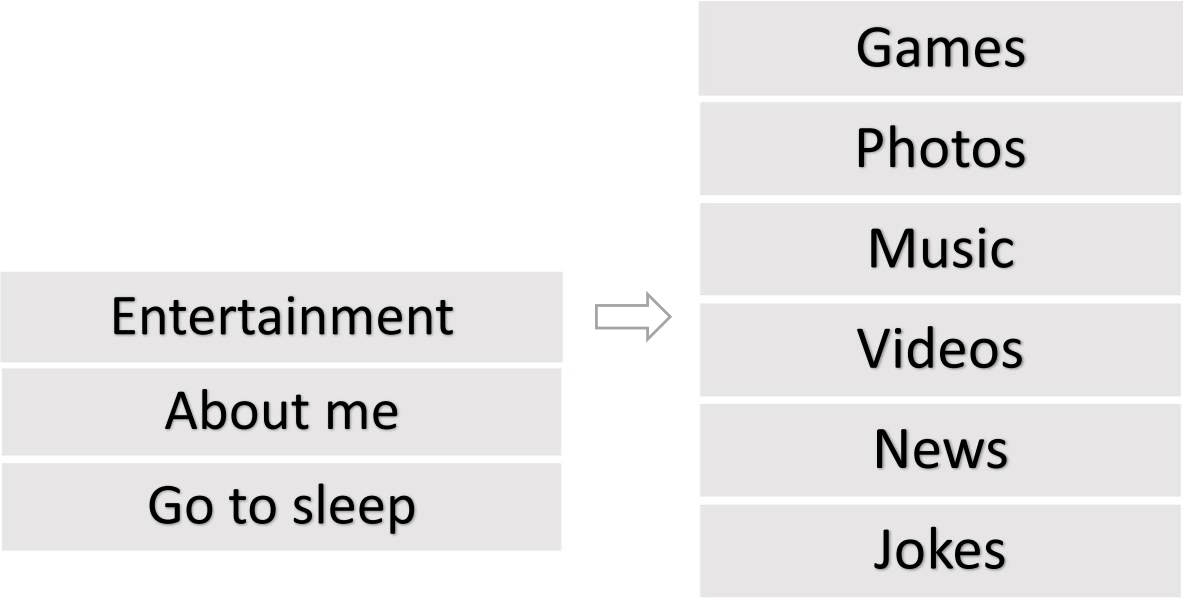
The tablet user interface

Figure 3 demonstrates the user interface and applications list through which the user will interact with MINI.

### Resources

The Intras Foundation in Zamora, Spain, will provide this study with a research lab as the research setting. The University of Carlos III of Madrid will provide the MINI robot and take the responsibility for technical support of the system. Human resources and materials will be supplied by the Memory clinic of Intras foundation in Zamora (IBIP). Other collaborating centers will be the Hospital Provincial of Zamora and the University of Salamanca, Salamanca, Spain.

### Measurements

#### Acceptance and usability

The acceptance and usability will be measured using a survey questionnaire based on the *Almere Model* by Heerink [17] (Supplementary file 1). Areas of measure are as follows: Anxiety, trust, attitude, appearance of the robot, personality of the robot/ social presence, perceived enjoyment, ease of use, sociability, adaptability, and usefulness, as well as, specific functionalities of the robot. The relevant statement has been produced by the researchers (AM, JMT) for each area. A five-point Likert scale (Strongly Disagree = 1, Disagree = 2, Neutral = 3, Agree = 4, Strongly Agree = 5) will be utilized to score each statement. We will also measure usability by the well-developed System Usability Scale (SUS) [22] which consists of a 10-item questionnaire with a five-point Likert scale (Strongly Disagree = 1, Disagree = 2, Neutral = 3, Agree = 4, Strongly Agree = 5) five response options for respondents; from Strongly agree to Strongly disagree (Supplementary file 2).

### Observed emotions

The observed emotions will be measured through the Observed Emotion Rating Scale (OERS) [23] (Supplementary file 3). Previously named the *Apparent Affect Rating Scale*, OERS is an observational tool for rating two positive emotions (pleasure and general alertness) and three negative emotions (anger, anxiety or fear, and sadness). Each item is accompanied by a list of the symptoms associated with that emotion and a drawing of a face demonstrating that emotion. The degree to which each emotion is expressed during a ten-minute observation period is rated on a scale of 1 (never) to 5 (always) (more than 5 min).

### Qualitative measurements

Semi-structured physical and video observations of participants’ emotions and interactions will be conducted by researchers. Besides, individual interviews with participants will be organized to obtain in-depth information on their experiences and attitudes towards the robot. The interview questions are presented in Table 1.

**Table 1:** Interview Questions

1.	What do you think about MINI? (e.g., appearance, feelings, the experience of using MINI)
2.	What does MINI do for you?
3.	Does MINI help you feel better?
4.	Is there anything you don’t like about MINI?
5.	Is there any other game/ exercise you would like to do with MINI?
6.	I gave you MINI for 30 mins every session. Was this too long or too short? Was 2–3 days a week too frequent or not enough?
7.	Is there anything you would like to change about MINI?
8.	Is there anything else you would like to tell us about MINI?

### Analysis

#### Quantitative data

Statistical analysis of the data will be performed through IBM SPSS v.24. Descriptive statistics of demographic and personal data will be presented and compared between groups. After the analysis of normal distribution and homogeneity of data, the repeated measures analysis of variance (ANOVA) will be conducted for pre-and post-intervention outcomes. If the distributional assumption were not satisfied nonparametric methods (Mann–Whitney U test, Kruskal-Wallis analysis of variance, Wilcoxon signed-rank test, calculation of Spearman’s correlation coefficient, etc.) will be applied.

#### Qualitative data

Thematic analysis will be performed for the data obtained from the focused group interviews employing Microsoft excel by the six-step approach of Braun and Clarke [24] as follows; 1) become familiar with data, 2) Generate initial codes, 3) Search for themes, 4) Review themes, 5) Define themes, 6) Write-up. After the interviews are transcribed, the coding of the data will be performed by Nvivo 12 software and the result will be thematically presented. Tables 2 and 3 demonstrate the timetable of the quantitative and qualitative evaluations and projects, respectively.

**Table 2:** Assessments

Activity/Assessment	Staff member	Time	Pre-study	Pre-study baseline	1	2
MMSE			X			
Acceptability questionnaire				X		
OESR				X	X	X
Interview						
Observation of interactions				X	X	X

**Table 3:** Project Timeline

Activity/month	Responsible	3	2	1	0	1	2	3
Project development	Primary researchers	X	X	X				
Ethical committee approval	Primary researchers			X				
Protocol preparation	Primary researchers	X	X	X				
**Acceptability and usability study (AU-S)**				X	X	X	X	
Participant’s selection	Researchers responsible for AU-S			X				
Informed consent	Researchers responsible for AU-S			X				
User’s testing	Researchers responsible for AU-S			X				
Incidence registration	Researchers responsible for AU-S				X	X	X	
**Efficacy study**						X	X	X
Participants selection	Responsible for interviewers			X				
Pre-evaluation	Interviewers				X			
Initialization session	Responsible for the study				X			
Intermediate evaluation	Responsible for the study				X	X	X	
Post evaluation	Interviewers						X	
Interview	Responsible for the study						X	
**Data management and result**		X	X	X	X	X	X	X
Database development	IBIP			X	X			
Data collection	Responsible for the study			X	X	X	X	
Data analysis	IBIP, USAL, UC3M						X	
Results and conclusion	Primary researchers						X	
**Dissemination**		X	X	X	X	X	X	X
Dissemination of results (Publications, conference presentation)	Primary researchers, UC3M						X	

## Discussion

This study will be an important contribution to the field of social robots in dementia care. Our study will be the first usability and acceptability trial of the MINI robot in a research lab with PwD and MCI. By using the Almere Model, the most complete acceptance model, observations, and interviews, it is intended that this study would capture the most significant aspects of usability and acceptability. With the aid of the trial’s findings, the designers, developers, and researchers of the robot will be able to further refine the MINI robot for the next extensive efficacy and implementation.

The small-scale efficacy study will be valuable in examining the effects of the MINI robot on immediate positive and negative emotions while live interactions. Positive emotions and reduced negative emotions during the interaction by enabling a meaningful engagement with the MINI robot will support the overall social health and wellbeing of PwD and MCI.

## Supplementary Information

**Additional file 1.** Almere model base questionnaire for Acceptability.

**Additional file 2.** System Usability Scale.

**Additional file 3.** Observed Emotions Rating Scale.

## Data Availability

Not applicable.

## Abbreviations

AAL: Ambient assisted living
ANOVA: Analysis of variance
ICT: Information and communication technology
MCI: Mild cognitive impairments
OERS: Observed affects and emotions
PwD: People with dementia
UTAUT: Unified theory of acceptance and use of technology
