# Supplementary material for: The usability and feasibility validation of the social robot MINI in people with dementia and mild cognitive impairment; a study protocol
Source: BMC Psychiatry. 2022 Dec 5;22:760. doi: 10.1186/s12888-022-04418-9 (PMC9720935; doi:10.1186/s12888-022-04418-9)
Supplement: Supplementary file 1 — Additional file 1. Almere model base questionnaire for Acceptability. [file 12888_2022_4418_MOESM1_ESM.pdf]

ALMERE MODEL (Acceptability)

		5<br>Completely agree	4<br>Relatively agree	3<br>Neutral	2<br>Somewhat disagree	1<br>Completely disagree
1	When using the robot I have been afraid of making mistakes with it.					
2	When using the robot I was afraid of breaking something					
3	The robot scares me					
4	I think it's a good idea to use the robot.					
5	I would like to have the robot at home					
6	If I had the robot at home I would use it often					
7	I think the robot can be adapted to what I need					
8	I think the robot will only do what I need at a time when particular					
9	I think the robot will help me when I need it					
10	I like that the robot talks to me.					
11	I like doing things with the robot.					
12	I find the robot nice.					
13	The robot seems boring to me					
14	I think I will quickly know how to use the robot					
15	The robot seems easy to use					
16	I think I can use the robot without help					
17	I think I can use the robot when someone is around to help					
18	I find the robot a pleasant conversation partner					
19	I find it pleasant to interact with the robot					
20	I feel that the robot understands me					
21	I think the robot is cute					
22	I think the robot is useful to me					
23	I should have the robot					
24	I think the robot would be good entertainment					
25	I feel like the robot has life					
26	I feel that the robot is not a real person					
27	Sometimes the robot seems to have real feelings					
28	The robot inspires me confidence					
29	The robot seems smart to me					
