# Supplementary material for: The usability and feasibility validation of the social robot MINI in people with dementia and mild cognitive impairment; a study protocol
Source: BMC Psychiatry. 2022 Dec 5;22:760. doi: 10.1186/s12888-022-04418-9 (PMC9720935; doi:10.1186/s12888-022-04418-9)
Supplement: Supplementary file 2 — Additional file 2. System Usability Scale. [file 12888_2022_4418_MOESM2_ESM.pdf]

Participant ID:	Place:	Date://

System Usability Scale

Instructions: For each of the following statements, please mark the response that best describes your reactions to the robot today.

	Fully disagree	Relatively disagree	Neutral	Relatively agree	Completely agree
1. I think I would like to use this robot often	1	2	3	4	5
2. I found the robot unnecessarily complex	1	2	3	4	5
3. I thought the robot was easy to use	1	2	3	4	5
4. I think that I would need the support of an expert to use the robot	1	2	3	4	5
5. I found the various possibilities of the robot quite well integrated	1	2	3	4	5
6. I thought there was too much inconsistency in the robot	1	2	3	4	5
7. I imagine most people would learn very quickly to use the robot	1	2	3	4	5
8. I found the robot very uncomfortable to use	1	2	3	4	5
9. I felt very confident in handling the robot	1	2	3	4	5
10. I need to learn many things before handling the robot	1	2	3	4	5

Please provide some comments about the robot:

This questionnaire is based on the System Usability Scale (SUS), which was developed by John Brooke while working at Digital Equipment Corporation. © Digital Equipment Corporation, 1986.
