# Supplementary material for: The usability and feasibility validation of the social robot MINI in people with dementia and mild cognitive impairment; a study protocol
Source: BMC Psychiatry. 2022 Dec 5;22:760. doi: 10.1186/s12888-022-04418-9 (PMC9720935; doi:10.1186/s12888-022-04418-9)
Supplement: Supplementary file 3 — Additional file 3. Observed Emotions Rating Scale. [file 12888_2022_4418_MOESM3_ESM.pdf]

Polisher Research Institute
Abramson Center for Jewish Life
(formerly the Philadelphia Geriatric Center)

Observed Emotion Rating Scale

M. Powell Lawton
Kimberly Van Haitsma
Jennifer Klapper

A Note on the Use of this Scale:

M. Powell Lawton, Ph.D., former director of the Polisher Research Institute, died in 2001. It was Dr. Lawton's intent that his scales be made widely available, and he wanted them to be in the public domain. While the Polisher Research Institute owns the copyright to this scale, permission is granted to use or reproduce his scales, provided that proper attribution is given.

This scale was first published as the Philadelphia Geriatric Center Affect Rating Scale: Lawton, M.P., K.S. Van Haitsma, and J.A. Klapper. " Observed Affect in Nursing Home Residents." Journals of Gerontology B : Psychological Sciences, 51:1(1996), 3-14.

A revised version was later published as the Apparent Affect Rating Scale:
Lawton, M.Powell; Van Haitsma, Kimberly; Perkinson, Margaret; Ruckdeschel, Katy. "Observed Affect and Quality of Life in Dementia: Further Affirmations and Problems" *Journal of Mental Health and Aging* 5:1(1999), 69-81.

While the latter scale is the same as the scale included on our website, it is now known as the Observed Emotion Rating Scale. We suggest the following citation:
Lawton, M.P., K. Van Haitsma, J.A. Klapper. (1999) Observed Emotion Rating Scale. Retrieved [*insert date*] from www.abramsoncenter.org/PRI (scales page).

Authors who wish to reproduce part of an article where a scale first appeared should request permission from the journal publishers before doing so.

Lastly, it would be appreciated if a reprint of any resulting articles be forwarded to the Research Librarian, or if a book is published, that purchasing information be provided to the Research Librarian.

OBSERVED EMOTION RATING SCALE

RESIDENT'S NAME ________________ UNIT: _________ OBSERVER'S NAME: __________________ DATE: TIME:

Please rate the extent or duration of each affect over a ten-minute period. Some possible signs of each emotion are listed.
If you see no sign of a particular feeling, rate "Never."

		7	1	2	3	4	5
		Not in view	Never	Less than 16 sec.	16-59 sec.	1-5 min.	more than 5 min.
PLEASURE<br>Signs: Laughing; singing; smiling; kissing; stroking or gently touching other; reaching out warmly to other; responding to music (**only** counts as pleasure if in combination with another sign).	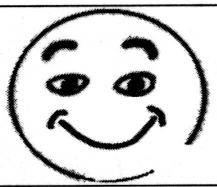						
ANGER<br>Signs: Physical aggression; yelling; cursing; berating; shaking fist; drawing eyebrows together; clenching teeth; pursing lips; narrowing eyes; making distancing gesture.	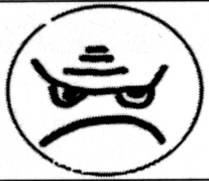						
ANXIETY/FEAR<br>Signs: Shrieking; repetitive calling out; restlessness; wincing/grimacing; repeated or agitated movement; line between eyebrows; lines across forehead; hand wringing; tremor; leg jiggling; rapid breathing; eyes wide; tight facial muscles.	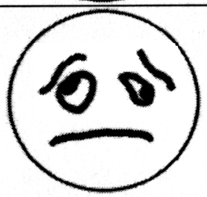						
SADNESS<br>Signs: Crying; frowning; eyes drooping; moaning; sighing; head in hand; eyes/head turned down and face expressionless (**only** counts as sadness if paired with another sign).	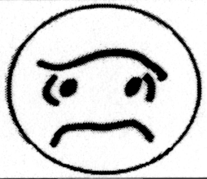						
GENERAL ALERTNESS<br>Signs: Participating in a task; maintaining eye contact; eyes following object or person; looking around room; responding by moving or saying something; turning body or moving toward person or object.	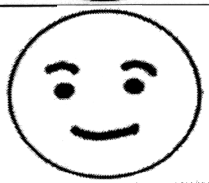						
